# Fluorouracil implants caused a diaphragmatic tumor to be misdiagnosed as liver metastasis: a case report

**DOI:** 10.1186/s12885-016-2778-z

**Published:** 2016-09-26

**Authors:** Yang-Yang Shen, Hong-Wei Qin, Jian-Bo Zhang, Zhen-Dan Wang, Pang Li, Kai Pang, Bo Zhang, Sheng Li, Kai Cui

**Affiliations:** 1 Shandong Cancer Hospital affiliated to Shandong University, Shandong, 250117 People’s Republic of China; 2 Shandong Academy of Medical Sciences, Jinan, Shandong 250200 People’s Republic of China; 3 Shandong Academy of Agricultural Sciences, Institute of Agricultural Quality Standards and Testing Technology, Jinan, Shandong 250100 People’s Republic of China; 4 Optoelectronics Technology Co., Ltd. Shandong Ruihua TONGHUI, Jinan, Shandong 250022 People’s Republic of China; 5 Shandong Provincial Collaborative Innovation Center for Neurodegenerative, DisordersQingdao University, Qingdao, Shandong 266071 People’s Republic of China

**Keywords:** Fluorouracil implants, Complication, Liver metastases, Raman spectroscopy, Sinofuan

## Abstract

**Background:**

Fluorouracil implants are widely used in peritoneal interstitial chemotherapy. Curative effects have been obtained, but implants have also caused some complications.

**Case presentation:**

We performed an analysis of a 66-year-old male patient’s case history, as well as conventional pathological analysis and Raman spectroscopic detection of the diaphragmatic tumor. We also analyzed the underlying causes of this condition to prevent complications and reduce misdiagnoses in future cases. The patient had a history of peritoneal fluorouracil implantation. Pathological analysis of the diaphragmatic mass revealed foreign particles, and Raman detection showed that the mass contained fluorouracil.

**Conclusion:**

Fluorouracil implants may persist due to the high concentrations of this drug used in peritoneal chemotherapy. This finding should provide guidance and improve the application of peritoneal implants. In clinical trials, and the diagnosis of liver metastasis should be based on pathological results.

## Background

Since the 1990s, carmustine implants have been used for glioma, and they were approved by the US Food and Drug Administration (FDA) for marketing in 1996 [1]. In China, the first sustained-release anti-tumor implant product approved by the CFDA was a polymer carrier containing 5- fluorouracil components named Sinofuan (Medicines NO. H20030345, Patent No. 1028685) [2]. Compared with intravenous fluorouracil chemotherapy, fluorouracil implants such as Sinofuan can improve the concentration, extend the effective time, and reduce the toxicity of chemotherapy. Moreover, these implants allow for targeted effects. Since 2003, fluorouracil implants have been widely used in peritoneal interstitial chemotherapy for gastric [3], colorectal [4, 5] and ovarian cancers [6]. They has also been widely used in percutaneous tumor implants for liver cancer [7]. However, this application has had significant effects, with related complications including anastomotic fistula, intestinal obstruction, chemical peritonitis, and wound infection [8]. The case presented here involves a high concentration of fluorouracil implants that gathered in the diaphragm, resulting in local tissue necrosis and fibrous lumps that oppressed the liver.

## Case presentation

**Fig. 1:**
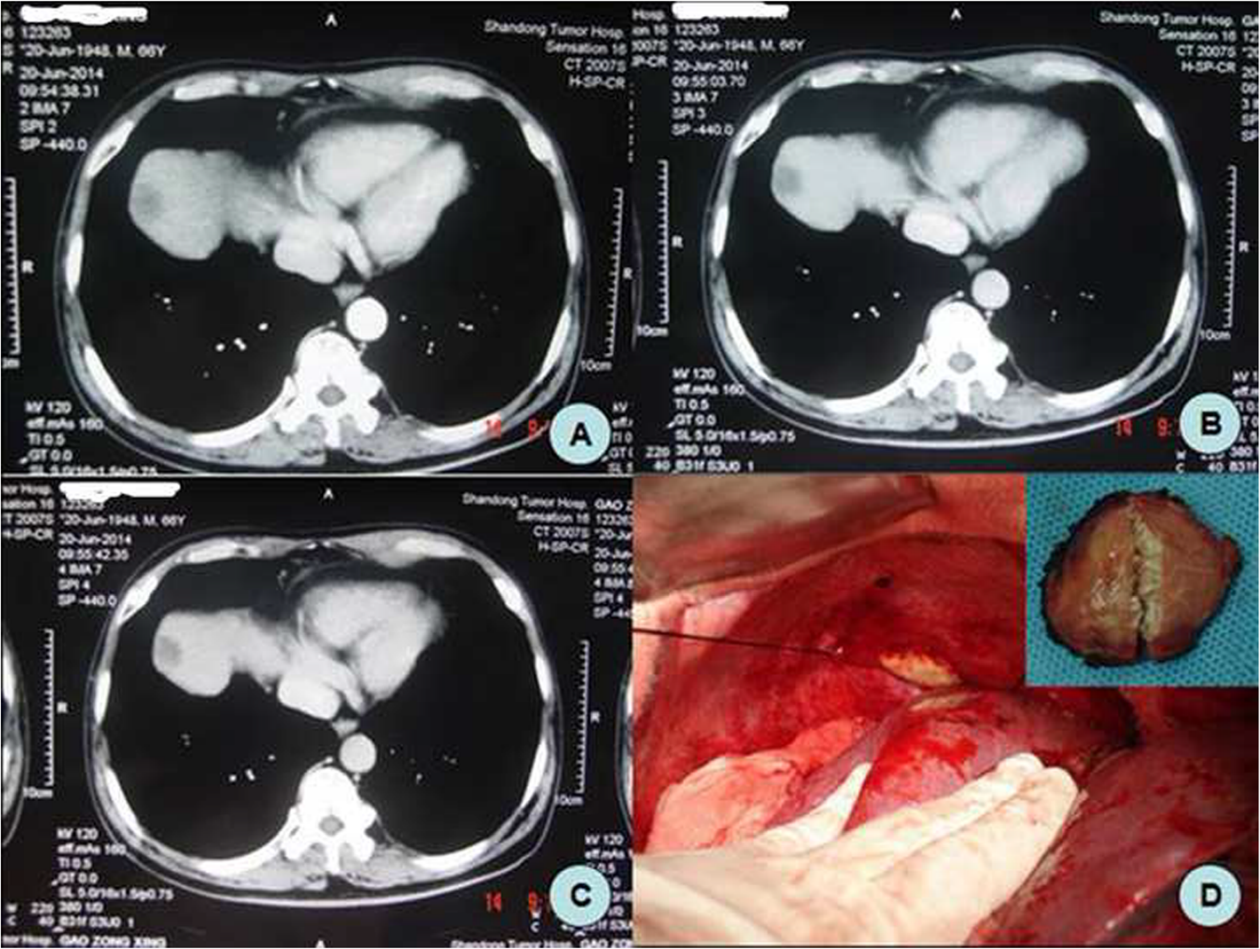
**a** Arterial phase (09:54:38.31), **b** Portal venous phase (09:55:03.70), **c** Venous phase (09:55:42.35), **d** Diaphragmatic tumor

**Fig. 2:**
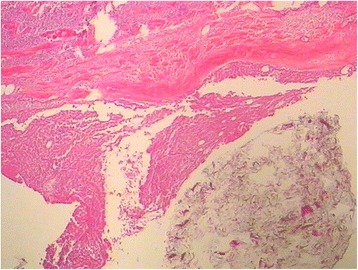
Diaphragmatic tumor (hematoxylin and eosin, magnification × 100)

**Fig. 3:**
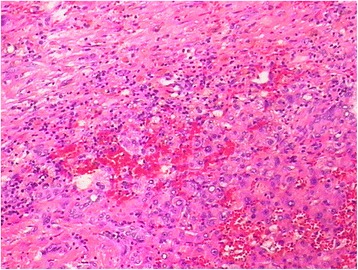
Hepatic nodule (hematoxylin and eosin, magnification × 100)

A 66-year-old male patient was diagnosed with sigmoid colon cancer by colonoscopy 3 months previously. No neoadjuvant therapy was done before surgery. The surgery consisted of sigmoid colon cancer resection performed at Qilu Hospital of Shandong University. It was an open operation. When exploring the abdomen, liver and peritoneum, no metastatic nodules were found, but a tumor, about 5 × 3 cm in size, was located in the sigmoid colon. The tumor was removed, an anastomosis was done at the rectum end of the sigmoid colon and plasma muscularis sutures embedded the anastomosis. 800 mg Sinofuan was planted in the abdomen for adjuvant chemotherapy, and a drainage tube was installed to protect the anastomosis. The operation went smoothly. Postoperatively, the patient presented abdominal pain, fever, and abdominal fluid. Encapsulated fluid measuring approximately 6.3 cm × 3.4 cm was detected in the right upper abdominal cavity, appearing as an echo-free space on ultrasonic screening. After paracentesis drainage and anti-infection treatment, the patient’s condition improved and he was discharged. One month ago, the patient came to our hospital for further chemotherapy. Physical examination showed no abnormalities. Computed tomography (CT) examination revealed a right liver lobe lesion of lower density on the eighth segment, with a diameter of approximately 2.0 cm. On CT the arterial phase, portal venous phase, and venous phase showed no obvious enhancement. However, the patient had normal levels of alpha-fetoprotein (AFP), carcinoembryonic antigen (CEA), cancer antigen 19-9 (CA19-9), gamma-glutamyltransferase (GGT), and lactate dehydrogenase (LDH). Before the colon cancer surgery they were also at normal levels. Combined with the history of colon cancer surgery and the CT manifestations, this case was initially diagnosed as liver metastasis. After three rounds of chemotherapy, CT showed no significant changes compared with the previous examination. The patient was then transferred to the surgical ward for further surgery. During surgery, a 3 cm × 2 cm × 0.7 cm encapsulated white porcelain-like tumor was detected on the diaphragm oppressing the liver, which caused liver capsule depression with local shallow sclerosis (see Fig. 1). The pathological results (see Figs. 2 and 3) showed the diaphragmatic surface of the tumor to be highly necrotic, oozing, and containing cylindrical foreign bodies. In addition, there was nodular surface hemorrhaging and necrosis, subcapsular inflammatory cell infiltration, fibrosis, and small bile duct hyperplasia.

**Fig. 4:**
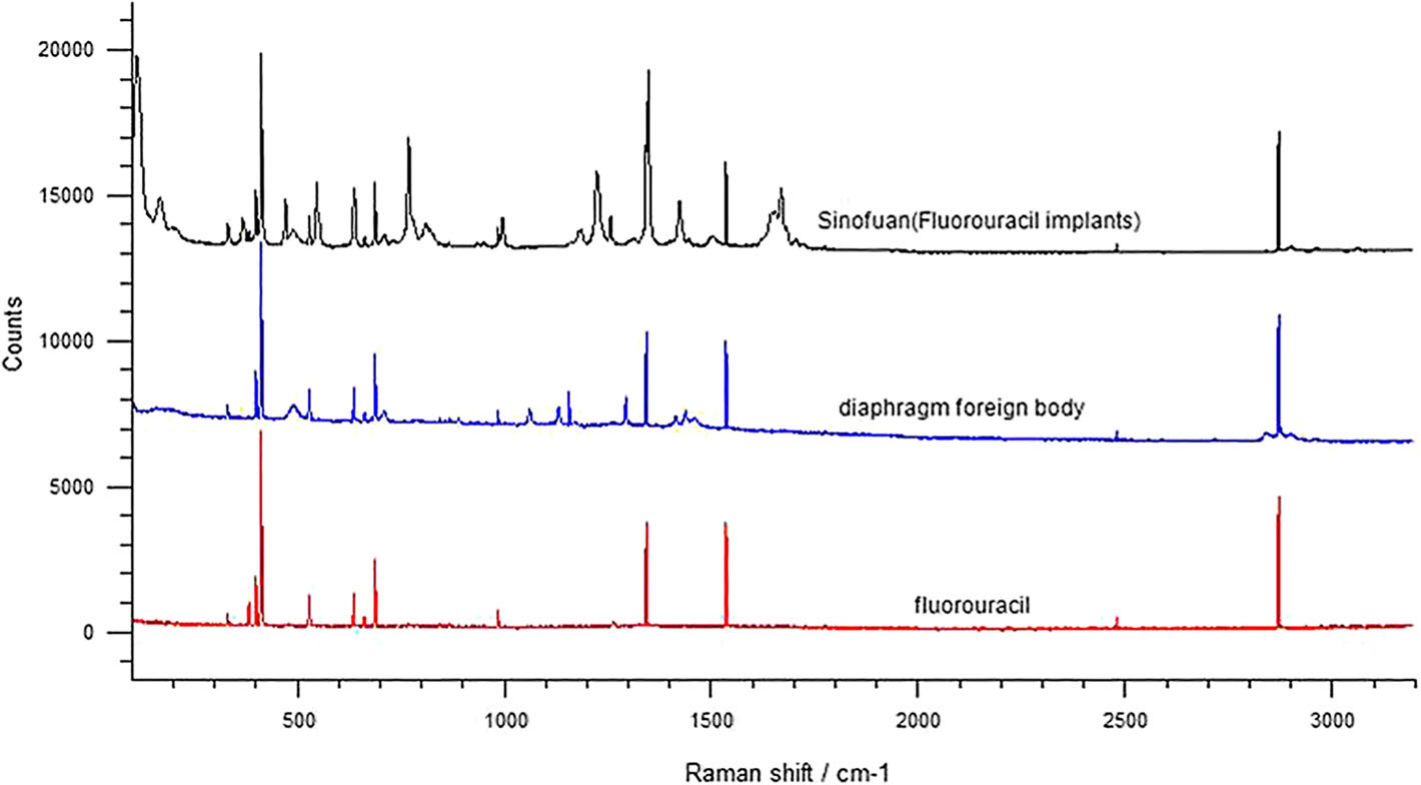
Raman spectra of fluorouracil, the diaphragmatic foreign body and Sinofuan

The Invia confocal Raman microscope by Renishaw was used to test the remaining foreign bodies in the diaphragm. A 785-nm wavelength laser was used and focused by an objective lens with 50X magnification. The irradiation power was set to 1 %, and the integration time was 10 s. The collection range of the spectrum was from 500 to 3200 cm^−1^. Samples included the cylindrical foreign body which was embedded in pathological wax blocks, Sinofuan (Wuhu harbinger of human medicine, Product lot NO.20130202), and the fluorouracil injection (HAIPU, Product lot NO.FA140506). WIRE4.0 spectral software (see Fig. 4) was used for analysis. The three spectra all showed significant peaks in the areas of 412, 687, 1344, 1538, and 2876 cm^−1^ of roughly the same height. Thus, it was concluded that both the cylindrical foreign body in the pathological wax blocks and Sinofuan contained fluorouracil.

## Discussion

Local recurrence and distant metastasis especially liver and pulmonary are common in colon cancer. In 50 % of colorectal cancer patients an increase of CEA is a signal of recurrence after tumor resection and 15 % of colorectal tumors do not release CEA [9]. In the case described here, the patient had a history of colon cancer surgery, and the atypical CT performance for each period of the liver was due to the low density of the tumor (see Fig. 1). Tumor markers such as CEA showed no abnormalities closely associated with the diaphragm, and focal puncture pathology was not easy to achieve. All these resulted in misdiagnosis as liver metastasis. Surgery should have been performed when the tumor appeared as a single and small mass on CT. But because of the patient’s physical condition and quality of life, he chose to first have chemotherapy.

In this case, the center of the diaphragmatic mass showed some white porcelain-like cylindrical particles (see Fig. 1). The patient had received an 800 mg fluorine surgical implantation. Based on the surgical, pathological, foreign body shape, and Raman spectroscopy results (see Fig. 3), the cylindrical particles were identified as non-degraded fluorouracil implants.

In clinical practice, abdominal foreign bodies become fiber-wrapped due to inflammation caused by the formation of lumps, and misdiagnosis is not uncommon [10]. Items such as legacy gauze [11], surgical sutures [12], and fish and chicken bones penetrating into the abdominal cavity [13, 14] have formed tumors.

Intraperitoneal chemotherapeutic drugs such as epirubicin, mitomycin, and cisplatin are in the experimental stage, and there have been no reports of complications such as in this case. In this case, the foreign body was aggregated Sinofuan. These implants, which should be biodegradable, gathered in the diaphragm due to their high concentration, causing local tissue necrosis and becoming fiber wrapped to cause liver tumor oppression. This situation was unlike anastomotic bleeding, abdominal aortic hemorrhage [15], liver granuloma [16], and other rare complications due to the uneven spread in surgery. Because of ascites after surgery, Sinofuan gathered in the diaphragm. Qin ZG et al. [17] conducted experiments in mice using Sinofuan administered intraperitoneally and found that after a period of implantation, non-degraded polymers crowded around the liver, the gap between liver, and stomach and the diaphragm. These findings reveal that Sinofuan has some risk of spreading after surgery.

## Conclusions

This report demonstrates a new, rare complication of Sinofuan. Our findings suggest that high-concentration fluorouracil implants can cause local tissue necrosis and fiber-wrapped formations. Therefore, the application of fluorouracil implant surgery must be improved. In particular, Sinofuan should be used during the preoperative period under strictly controlled indications [2], such as (1) in patients at high risk of colorectal cancer recurrence, especially serious tumor invasion to adjacent tissues. (2) In those with lymph node metastasis or malignant ascites. (3) In cases with positive or suspicious peritoneal lavage cytology, those when high levels of CEA and CK20 molecules in the peritoneal rinse are positive, or when there is suspicion of micrometastasis. (4) In tumors causing excessive compression during tumor removal surgery and cases with ruptured intestines. The contraindications for fluorouracil implantation include tumor perforation with peritonitis, intestinal obstruction, severe anemia, hypoproteinemia, and abundant ascites [2]. Patients should be evaluated preoperatively for nutritional status and liver function. Moreover, these implants should be spread as evenly as possible or fixed with biological glue. The patient’s condition should also be carefully monitored, and fluorouracil implants should be avoided in patients prone to postoperative issues.

## Abbreviations

AFP: Alpha-fetoprotein
CA19-9: Cancer antigen 19–9
CEA: Carcinoembryonic antigen
CFDA: China Food and Drug Administration
CK20: Cytokeratin 20
CT: Computed tomography
FDA: Food and Drug Administration
GGT: Gamma-glutamyltransferase
LDH: Lactate dehydrogenase
